# Risk of autism spectrum disorder in children with a history of hospitalization for neonatal jaundice

**DOI:** 10.3906/sag-2103-263

**Published:** 2021-10-21

**Authors:** Gaffari TUNÇ, Ayla UZUN ÇİÇEK, Fatih KILIÇBAY

**Affiliations:** 1 Department of Neonatology, Faculty of Medicine, Sivas Cumhuriyet University, Sivas Turkey; 2 Department of Child and Adolescent Psychiatry, Faculty of Medicine, Sivas Cumhuriyet University, Sivas Turkey

**Keywords:** Newborn, autism spectrum disorder, neonatal jaundice, phototherapy

## Abstract

**Background/aim:**

Limited research has focused explicitly on the association between neonatal jaundice and autism spectrum disorder (ASD), and inconclusive evidence exists in the literature within this framework. This study aimed specifically to investigate whether neonatal jaundice is a potential risk factor for ASD and whether there is a connection between the types of neonatal jaundice and the severity of ASD.

**Materials and method:**

This study involved 119 children with ASD [90 males (75.6%), 29 females (24.4%), mean age: 45.39 ± 11.29 months] and 133 healthy controls [100 males (75.2%), 33 females (24.8%), mean age: 46.92 ± 11.42 months]. Psychiatric disorders were diagnosed through the Diagnostic and Statistical Manual of Mental Disorders criteria. Childhood Autism Rating Scale (CARS) was used to assess the screening and diagnosis of autism. A specially prepared personal information sheet was employed to investigate socio-demographic characteristics and birth and clinical histories.

**Results:**

The rate of the history of jaundice and pathological jaundice requiring hospitalization and phototherapy were significantly higher in the ASD group compared to the controls. CARS total score and the mean scores of nearly all items were statistically higher in children with a history of pathological jaundice than those with a history of physiological jaundice.

**Conclusion:**

Neonatal jaundice, depends on its severity, seems to be one of the possible biological factors associated with subsequent development of and the severity of ASD. Establishing a causal relationship between neonatal jaundice and ASD by more comprehensive studies may contribute to alleviating of the severity of ASD for individuals at risk.

## 1. Introduction 

Autism spectrum disorder (ASD) is a group of neurodevelopmental disorders characterized by impairments in social communication and interaction, restricted and repetitive patterns of behaviors that can persist throughout life [1]. Although many risk factors are proposed, unfortunately, the specific causes of ASD still remain to be elucidated, and environmental and/or genetic factors are among the main contributing factors [2]. In fact, there is strong evidence that perinatal and obstetric events, which are among the investigated biological and environmental risk factors, are involved in the pathogenesis of ASD. Previous studies have emphasized the importance of prematurity, cesarean delivery, low Apgar score, low birth weight, and hypoxia among perinatal risk factors [2,3]. However, researchers investigating the relationship of perinatal risk factors and autism phenotypes have stressed that many of these factors are not the predominant cause even though they are implicated in autism risk and form part of the epiphenomena of genetic autism disposition [3,4].

Neonatal jaundice is one of the perinatal events that may play a potential risk factor for autism. Neonatal jaundice is one of the most prevalent clinical conditions, with being observed in approximately 60% of term and 80% of preterm neonates in the first week of life and a normal transitional phenomenon that typically resolves without any treatment for most neonates. However, it is also the leading reason for newborn hospital readmissions worldwide [5]. Hyperbilirubinemia in neonates has been classified as physiological and pathological jaundice [6]. The most common causes of pathological jaundice in the neonatal period are hemolytic jaundice, ABO incompatibility, rhesus incompatibility, G6PD deficiency, dehydration due to lack of proper feeding, breast milk jaundice, cephal hematoma, and polycythemia [7]. Actually, unconjugated bilirubin is beneficial at a low level. It functions as a potent antioxidant agent, while, at higher concentrations, it can cross the intact blood-brain barrier and be neurotoxic in the developing brain of newborns [8–10]. Therefore, unconjugated hyperbilirubinemia in the neonatal period is of concern since it can be related to lifelong sequelae and poor developmental and neurocognitive outcomes, including cerebral palsy, intellectual disability, brain dysfunction, attention deficit hyperactivity disorder, and several impairments of language and communication similar to ASD [8,11,12].

Previous studies exploring the relationship between the history of neonatal jaundice and ASD have produced conflicting results. While some studies have indicated that infants who developed neonatal jaundice are subsequently at risk of ASD [13–18), others have failed to find any significant association [19–21]. Moreover, limited research has focused mainly on the association between the history of neonatal jaundice and ASD. Since there is no conclusive data about the relationship between previous neonatal jaundice and the development of ASD in childhood, we thought this might be a relevant risk factor for ASD. The aim of current study was to assess the risk of autism spectrum disorder in children with a history of hospitalization for neonatal jaundice.

## 2.Material and methods

### 2.1.Participants 

This cross-sectional study was carried out in 119 children with ASD [90 males (75.6%), 29 females (24.4%) with a mean age 45.39±11.29 months] and 133 healthy controls [100 males (75.2%), 33 females (24.8%) with a mean age 46.92±11.42 months] who matched for sex, age, and socio-cultural characteristics with the ASD group. Children with ASD admitted to the Child and Adolescent Psychiatry Outpatient Clinic were consecutively enrolled in the study. ASD was the primary diagnosis and the main reason for seeking treatment in the patient group. The control group was randomly recruited from healthy-normal children who applied to the general pediatric outpatient clinics. Pregnant women who are hospitalized for systemic infections and antibiotic treatment were considered as having severe infections during pregnancy. Seizure comorbidity was diagnosed in cases in which antiepileptic therapy was initiated by pediatric neurology. In this study, pathological jaundice was defined as hyperbilirubinemia requiring hospitalization for phototherapy at least 24 h, as per relevant national guidelines [7]. However, if a patient had had neonatal jaundice without requiring phototherapy on follow-up, it was considered to have physiological jaundice. Exclusion criteria were the history of stay in a neonatal intensive care unit, syndromic children, children with a history of bilirubin encephalopathy requiring exchange transfusion in the neonatal period, children with known chromosomal abnormalities, congenital anomalies, children with developmental delays or disorders due to other causes than ASD, and missing data on neonatal jaundice; children with adverse conditions such as extreme poverty and severe parental psychosocial deprivation; and macrosomic (n = 9), postterm (n = 8) cases. 

Each participant and his/her parents underwent a psychiatric examination based on the Diagnostic and Statistical Manual of Mental Disorders, Fifth Edition [1]. The study was carried out according to the Declaration of Helsinki and Good Clinical Practice procedures principles and approved by the local Ethics Committee of the Medical Faculty of the Sivas Cumhuriyet University (Date: 11.12.2019, No: 2019-12/20). The study’s aim and procedure were explained verbally and written, and verbal informed consent was obtained for each child.

### 2.2.Data collection tools 

#### 2.2.1.Personal information form 

The clinical data were collected as participants’ socio-demographic characteristics (age, sex, place of residence, family characteristics, etc.) and clinical data (participants’ prenatal/perinatal/neonatal history, health, and disease status, features of autism and neonatal jaundice, etc.) using a questionnaire specifically designed by the researchers. The diagnosis of neonatal jaundice was performed through information obtained from the parental interview and categorized into two groups as physiological and pathological jaundice. Children who were evaluated by pediatric psychiatry between the ages of 2 and 6 and diagnosed with autism spectrum disorder were also divided into two groups based on the history of physiological or pathological jaundice [7]. 

#### 2.2.2.Childhood Autism Rating Scale (CARS) 

CARS is a well-established instrument for the screening and diagnosis of autism. It consists of 15 items, which are scored on a 7-point Likert scale. The examiner/clinician assigns a score of 1 to 4 for each item: score 1 indicates behavior appropriate for age level, whilescore 2 shows mild, score 3shows moderate, score 4 shows severe deviance concerning normal behavior for age level, respectively. The items are “relating to people”, “imitative behavior,” “emotional response”, “body use”, “object use”, “adaptation to change”, “visual response”, “listening response”, “perceptive response”, “fear or anxiety”, “verbal communication”, “nonverbal communication”, “activity level”, “level and consistency of intellective relations”, and “general impressions”. A total score of ≥30 points indicates the presence of autism. The interval from 30 to 36.5 points describes mild to moderate autism, and scores of 37–60 define severe autism [22]. The scale’s validity and reliability have been demonstrated in Turkish children by Sucuoglu et al. [23].

### 2.3.Statistical analysis 

SPSS Statistics version 23 was used for statistical analysis. Kolmogorov–Smirnov test was performed to examine the distribution of data. The numerical and categorical data were presented as mean ± standard deviation (SD), number (n), median (min-max), and percentage (%) as appropriate. Potential risk factors associated with ASD were identified using univariate analysis. Categorical variables were compared using the chi-square or Fisher’s exact when appropriate, and continuous parametric variables were analyzed by the Student’s *t*-test or the Mann–Whitney test for continuous nonparametric variables. The variables with 2-tailed p-value of less than 0.05 in univariate analysis were included in a multivariate logistic regression model (stepwise) to evaluate the odds ratios and 95% confidence intervals to calculate the strength of any association. A *p*-value of less than 0.05 was considered statistically significant. 

## 3.Results

### 3.1.Socio-demographic and familial characteristics of the sample.

A total of 252 children, 119 with ASD and 133 controls, participated in this study. The ASD group and the control group did not differ significantly in terms of age, sex, the place of residence, family income level, parental education level, parental consanguinity, and maternal and paternal age at pregnancy (all p values >0.05). There was no significant difference between groups for the number of siblings (p = 0.387), but the proportion of being the first-born child was significantly higher in the ASD group (47.9% vs. 33.8%, respectively, p = 0.023). Besides, the presence of family history for ASD (39.5% vs. 6%, respectively, p < 0.001) was significantly higher in the ASD group than in the control group. The socio-demographic and familial characteristics are given in Table 1.

**Table 1:** Socio-demographic and familial characteristics of participants.

n (%)	ASD (n=119)	Controls (n=133)	p-value
Age (months)º	45.4±11.3	46.9±11.4	0.271
Gender (male)	90 (75.6)	100 (75.2)	0.935
Place of residence			0.402
Urban	82 (68.9)	85 (63.9)	
Rural	37 (31.1)	48 (36.1)	
Family income level			0.451
The minimum or less	42 (35.3)	41 (30.8)	
Above the minimum wage	77 (64.7)	92 (69.2)	
Maternal education level			0.906
Primary education and lower	42 (35.3)	46 (34.6)	
Upper primary education	77 (64.7)	87 (65.4)	
Paternal education level			0.808
Primary education and lower	42 (35.3)	45 (33.8)	
Upper primary education	77 (64.7)	88 (66.2)	
Parental consanguinity	28 (23.5)	34 (25.6)	0.708
Number of siblings (n%)			0.387
0	25 (21)	26 (19.5)	
1	55 (46.2)	58 (43.6)	
2	26 (21.8)	40 (30.1)	
≥3	13 (10.9)	9 (6.8)	
Maternal age at pregnancy (y)º	29.3±7.4	28.8±5.8	0.611
Paternal age at pregnancy (y)º	32.8±7.8	31.3±5.8	0.249
Family history of ASD	47 (39.5)	8 (6)	<0.001

ASD: Autism Spectrum Disorder, º:mean±SD, SD: Standard Deviation.

###  3.2.Maternal characteristics of participants.

Threatened miscarriage, preeclampsia, gestational diabetes, and other endocrine problems during pregnancy, ABO and/or Rh incompatibility, excessive tea and/or coffee consumption, radiation exposure, utilization of vitamins, minerals, and other supplements during pregnancy were similar between the two groups (all p values >0.05). Alcohol and/or substance use during pregnancy was not detected in the study group. On the other hand, the mothers of children in the ASD group were significantly more nulliparous than those in the control group (47.9% vs. 33.8%, respectively, p = 0.023). Also, the rate of smoking and severe infections during pregnancy was significantly higher in the mothers of children with ASD than those in the control group (p = 0.025, p = 0.048, respectively). Maternal characteristics of participants are shown in Table 2.

**Table 2:** Maternal characteristics of participants.

n (%)	ASD (n = 119)	Controls(n = 133)	p-value
Nulliparity	57 (47.9)	45 (33.8)	0.023
Threatened miscarriage	29 (24.4)	26 (19.5)	0.355
Preeclampsia	7 (5.9)	8 (6)	0.965
Gestational diabetes	9 (7.6)	4 (3)	0.103
Other endocrine problems other than diabetes during pregnancy	6 (5)	3 (2.3)	0.234
Severe infection in pregnancy	27 (22.7)	16 (12)	0.025
ABO and/or Rh incompatibility	5 (4.2)	4 (3)	0.610
Smoking during pregnancy	34 (28.6)	24 (18)	0.048
Excessive tea and/or coffee consumption during pregnancy	25 (21)	29 (21.8)	0.878
Radiation exposure in pregnancy	6 (5)	6 (4.5)	0.843
Vitamins, minerals, and supplements in pregnancy	93 (78.2)	101 (75.9)	0.677

ASD: Autism Spectrum Disorder

### 3.3.Perinatal and natal characteristics and some clinical features of participants

The use of vacuum-forceps was not detected in any of the cases. Children with ASD had a statistically higher percentage of being delivered by cesarean section (29.4% vs. 16.5%, p = 0.015). The rate of the history of seizures was significantly higher in the ASD group (35.3% vs. 3.8%, p < 0.001). The perinatal and natal characteristics of participants are shown in Table 3.

**Table 3:** Perinatal characteristics and some clinical features of participants.

n (%)	ASD (n=119)	Controls(n=133)	p-value
Season of birth			0.114
Winter (December-February)	23 (19.3)	31 (23.3)	
Spring (March-May)	29 (24.4)	36 (27.1)	
Summer (June-August)	45 (37.8)	32 (24.1)	
Autumn (September-November)	22 (18.5)	34 (25.6)	
Birth type			0.015
Vaginal delivery	84 (70.6)	111 (83.5)	
Cesarean section	35 (29.4)	22 (16.5)	
Gestational age at birth			0.149
Preterm (34-37 weeks)	21 (17.6)	15 (11.3)	
Term (≥37 weeks)	98 (82.4)	118 (88.7)	
Birth weight			0.277
<2500 g	19 (16)	15 (11.3)	
≥2500 g	100 (84)	118 (88.7)	
Birthplace			0.965
Hospital	112 (94.1)	125 (94)	
Others (house, car, etc.)	7 (5.9)	8 (6)	
Birth complication	14 (11.8)	8 (6)	0.106
Seizure/convulsion history	42 (35.3)	5 (3.8)	<0.001

ASD: Autism Spectrum Disorder.

### 3.4.Comparison of the jaundice features of the groups

The history of apparent jaundice in the ASD group was significantly higher than in the control group (55.5% vs. 41.4%, respectively, p = 0.025). Furthermore, the frequency of jaundice requiring hospitalization and phototherapy was significantly higher in the ASD group compared to the controls (25.8% vs. 10.9%, respectively, p = 0.038). In contrast, the percentage of exchange transfusion did not differ between the groups (p = 0.346). The data on the history of jaundice are given in Table 4.

**Table 4:** Comparison of the jaundice features of participants.

n(%)	ASD(n=119)	Controls(n=133)	p-value
History of jaundice	66 (55.5)	55 (41.4)	0.025
Jaundice types			0.038
Physiological	49 (74.2)	49 (89.1)	
Pathological	17 (25.8)	6 (10.9)	
Exchange transfusion	3 (2.5)	1 (0.8)	0.346

ASD: Autism Spectrum Disorder.

### 3.5.Multivariate analyses of variables associated with autism spectrum disorder

Univariate analyses of variables associated with the risk of development of ASD include a positive family history of autism, nulliparity, smoking during pregnancy, the experience of severe infection during pregnancy, delivery by cesarean section, history of neonatal jaundice, especially pathological jaundice, and the history of seizures. When these factors were placed into logistic regression analysis, it was found that the presence of positive family history for autism and the history of seizures were each independently associated with the risk of development of ASD in childhood (Table 5). 

**Table 5:** Multivariate analyses of variables associated with autism spectrum disorder.

Variable	Odds ratio	95% Confidence Interval		p
		lower	upper	
Nulliparity	1.56	0.66	3.66	0.308
Maternal smoking during pregnancy	2.38	0.92	6.18	0.074
Positive family history for autism	3.78	1.29	11.1	0.015
Cesarean delivery	1.59	0.64	3.99	0.322
The history of seizure	6.05	1.67	21.9	0.006
Neonatal jaundice	1.21	0.34	4.33	0.769
Severe infection in pregnancy	2.43	0.76	7.77	0.134

### 3.6.Comparison of clinical variables and CARS scores according to the type of jaundice

The presence of intellectual disability was not significantly affected by physiological or pathological jaundice types (p = 0.394). Still, the children with pathological jaundice had a substantially higher neonatal seizure history than the children with physiological jaundice (p < 0.001). As for the scores of the CARS scale according to the types of jaundice, CARS total score and the mean scores of all items except “fear or anxiety” in children with a history of pathological jaundice were statistically higher than the scores of children with a history of physiological jaundice (all p values <0.05). Data on the CARS scores by types of jaundice are shown in Table 6.

**Table 6:** Comparison of clinical variables and CARS scores by types of jaundice in participants.

mean ± SD	Physiological(n = 49)	Pathological(n = 17)	p-value
Presence of intellectual disabilityº	32 (65.3)	13 (76.5)	0.394
Seizure/convulsion historyº	17 (17.3)	14 (60.9)	<0.001
CARS-Relating to people	2.4 ± 1.2	3.06 ± 1.11	0.007
CARS-Imitative behavior	1.9 ± 1.0	2.34 ± 0.95	0.043
CARS-Emotional response	1.9 ± 1.0	2.58 ± 1.05	0.008
CARS-Body use	1.9 ± 0.8	2.30 ± 0.79	0.029
CARS-Object use	2.0 ± 0.8	2.43 ± 0.82	0.022
CARS-Adaptation to change	1.7 ± 1.5	2.13 ± 0.82	0.010
CARS-Visual response	1.9 ± 1.0	2.58 ± 1.05	0.010
CARS-Listening response	2.5 ± 0.5	2.82 ± 0.98	0.010
CARS-Perceptive response	1.5 ± 0.6	1.93 ± 0.77	0.008
CARS-Fear or anxiety	1.9 ± 0.7	1.89 ± 0.75	0.822
CARS-Verbal communication	2.6 ± 1.1	3.23 ± 1.00	0.006
CARS-Non-verbal communication	2.3 ± 1.3	3.00 ± 1.22	0.007
CARS-Activity level	2.4 ± 0.8	2.82 ± 0.96	0.036
CARS-Level and consistency of intellective relations	1.8 ± 0.9	2.65 ± 1.13	0.001
CARS-General impressions	2.1 ± 1.1	2.91 ± 1.18	0.001
CARS-Total score	30.3 ± 12.7	38.58 ± 12.77	0.003

CARS: Childhood Autism Rating Scale, º: n (%), SD: Standard Deviation.

## 4.Discussion

Given the effect of perinatal events on the developing brain, the current study aimed to assess whether neonatal jaundice may play a role in the development or the severity of childhood autism. The findings indicated that children with ASD have a significantly higher history of neonatal jaundice, especially pathological one, which also seems to affect the severity of the disease (higher CARS total score). However, it was found that neonatal jaundice is not independently a risk factor for ASD. On the other hand, the presence of positive family history for autism and the history of seizures seem to be associated with the development of ASD. 

Autism spectrum disorder is a heterogeneous disorder, and the specific cause of most cases of ASD remains unknown. However, a combination of genetic, developmental, and environmental factors are emphasized in its etiology [2,3,24,25]. As consistent with many previous studies, the presence of positive family history for autism emphasizes heritability of the disease, indicating the relevance of genetic factors in the pathogenesis. Numerous gene variations, which thought to affect the risk of developing ASD, have been reported, and most of them can interact with environmental factors to influence development in ways that cause to ASD. Although environmental factors are still poorly understood, numerous studies have identified some prenatal, natal, and postnatal risk factors, which have potential to contribute to cerebellar injury, abnormal brain development in ASD [2]. In general, the risk factors such as maternal mental and/or medical illnesses, younger maternal age, maternal age of >40 years old, birth complications, low birth weight, cesarean section, a low 5-min Apgar score, neonatal jaundice, smoking during pregnancy, and nulliparity have been proposed as drivers of increased risk [17,26]. Given that unconjugated hyperbilirubinemia occurs in varying degrees in newborn infants, especially in premature infants, during the first weeks of life, and having the potential to cause to cerebellar injury, hyperbilirubinemia has been questioned as one possible biological factor associated with subsequent development of ASD [17]. Metaanalyses have demonstrated that neonatal jaundice may be associated with ASD [16,17]. In fact, previous studies have generally together addressed environmental risk factors of autism; few studies have specifically investigated neonatal jaundice’s role on the risk of ASD. However, relevant research has resulted in inconsistent findings. Some studies demonstrated that neonatal jaundice increased the risk of ASD [12–18,27], whereas others did not [19–21]. A recent population-based study objecting to investigate long-term neurodevelopmental outcomes of significant neonatal jaundice reported a 1.5 to 2- times greater risk for ASD than the reference cohort [28]. Additionally, some studies have investigated premature (<37 weeks gestational age) and term infants with neonatal jaundice separately in terms of ASD risk and found a significant positive association in term infants but not in premature infants [13,29,30]. However, in contrast to those studies, it has been expected that premature infants would be more vulnerable to neurotoxic effects of bilirubin, and, therefore, it has been suggested that the measurements of bilirubin used by other methods may be better predictors for neurotoxicity in preterm infants. Additionally, contradictory findings in those studies may be caused by the studies’ methodology, such as the source of cases, the criteria used to identify both neonatal jaundice and ASD, designs of the studies, the age-group included, sex, or socio-demographic status. In the study presented here, because of that many premature infants experience intensive care support and may be exposed to many detrimental conditions during intensive care period, which most parents can’t know or remember, the patients with those criteria were not included to keep minimizing bias. 

In this study, it was revealed that the rate of every degree of neonatal jaundice, even physiological one, was significantly higher in the ASD group, suggesting a potential relationship between ASD and neonatal jaundice. Additionally, this study showed that the history of pathological jaundice was associated with higher CARS score, which represents clinical severity of the disease. Despite this association has been reported previously [14,26,27,31], the underlying mechanisms have not been clarified yet. Abnormal auditory evoked responses and impaired communication may be observed in both ASD and bilirubin-induced neurotoxicity, showing a potential temporal relationship [17]. On the other hand, many of the genes associated with ASD are also involved in the development of the brain [32], and, therefore, some proteins controlled by those genes may make the production, growth, or organization of neurons more vulnerable to neurotoxicity of bilirubin.

The potential brain damage due to hyperbilirubinemia has been suggested as the underlying mechanism resulting in ASD [15]. As it is well known, even though hyperbilirubinemia is almost always benign, it may also result in bilirubin-induced neurologic dysfunction at excessive levels. It can impact brain development, cognitive functions, synaptic plasticity, learning, and memory. The brain’s regions susceptible to bilirubin toxicity are the globus pallidus, hippocampus, peripheral and central auditory pathways, cerebellum, and subthalamic nuclear bodies [8,10]. Previous studies have proposed that crucial processes such as neurogenesis, myelination, and synaptogenesis during early brain development at these brain regions were negatively affected by bilirubin [10]. Many of these regions are also closely related to the pathogenesis of ASD [33,34]. It is well known that athetoid cerebral palsy, neuromotor abnormalities, neurobehavioral symptoms, intellectual disability, paralysis of upward gaze, sensorineural, audiological, and visual-motor dysfunctions, speech and communication impairments observed in kernicterus are similar to ASD [8,11,12,21,35]. However, these findings are the tip of the iceberg, and bilirubin-induced neurological dysfunction may also occur without characteristic clinical manifestations of apparent kernicterus [17,36]. Some authors have proposed that phototherapy might trigger the pathophysiological mechanisms of ASD through implicated in DNA damage and/or affecting cytokine levels and oxidative stress [37–39]. However, further researches should be attempted to establish a causal relationship between neonatal jaundice and ASD. If there is any impact of neonatal jaundice on the developing ASD, the prevention and early treatment of jaundice may be a promising step in reducing development or severity of ASD in individuals at risk, such as family members of a person with autism.

The present study findings demonstrate that family history of autism, nulliparity, maternal smoking, and severe infection during pregnancy, delivery by cesarean section, and the history of neonatal jaundice, in particular, pathological jaundice, and seizure are risk factors for ASD. Autism is more common in males, and, in this study, male sex made up three-quarters of patients in the ASD group. Because of age and sex-matched control group selection, male sex was not found as a risk factor in the statistical analyses. Multivariate analyses revealed that the presence of a family history of autism and seizure are independently associated with ASD. Epilepsy and autism may co-occur with the frequency of up to 20% of children with either disorder, suggesting that the two entities share underlying pathology. Despite it has not been clarified whether epilepsy contributes to ASD or is a consequence of ASD, growing evidence suggests that ASD is not the result of inadequately treated epilepsy [40]. The prevalence of ASD was higher in children with a history of seizures in the first year of life compared to the general population [41]. It has been reported that autistic children with epilepsy have more severe autism symptoms, and conversely, the risk of epilepsy is increased in ASD patients with higher scores [42]. Despite both diseases are etiologically heterogeneous, a possible relationship between epilepsy and ASD may be the result of abnormal brain development caused by neurobiological antecedent (structural or developmental lesions, genetic defects, or environmental insults) or abnormal neuronal plasticity [43]. Early detection and treatment of seizures may prevent severe symptoms of ASD. 

Given that most of the previous studies were based on ICD codes or insurance databases that might reveal treatment codes inconsistent with a definitive diagnosis, the strength of this study is the study population, which was selected from a relatively larger clinical sample. This study replicates and extends the previous studies’ results in children with ASD and puts some additional data on the relationship between neonatal jaundice and ASD. However, there are some limitations of this study. First, the study’s cross-sectional design and the sample enrollment from a single hospital prevent the generalization of the results and the determination of causality. Second, maternal interviews were used for the ascertainment method of neonatal jaundice. The diagnosis of jaundice was not confirmed by using the medical records of children; hence, the relationship between blood bilirubin concentrations and ASD was based on clinical jaundice. Further, “maternal interviews” may be prone to recall bias. Third, given the retrospective nature of the study, the data may not have included some necessary variables (such as the cause of the seizure, effects of drug or heavy metals, viral infections, genetics factors), and this might lead to the difficulty in evaluating temporal relationships between events. Finally, one of the limitations of the study is that it was not known exactly how many days of hospitalization for phototherapy in the neonatal period. 

## 5.Conclusion

This study showed that the rate of neonatal jaundice is higher in the patients subsequently suffering from ASD, and additionally it seems that jaundice requiring treatment affects the severity of ASD. Therefore, the prevention and early treatment of neonatal jaundice may be a promising step in reducing development or severity of ASD in individuals at risk. The presence of family history for ASD and the history of seizures seem to be independent risk factors for ASD. So, early detection and treatment of seizures appear to be important for minimizing the severity of ASD, which may develop later. However, more comprehensive, prospective studies with the objective of establishing a causal relationship between neonatal jaundice and ASD, as well as seizure and ASD, may provide evidence-based treatment strategies for patients with ASD. 

## Informed consent

The study was carried out according to the Declaration of Helsinki and Good Clinical Practice procedures principles and approved by the local Ethics Committee of the Medical Faculty of the Sivas Cumhuriyet University (Date: 11.12.2019, No: 2019-12/20). The study’s aim and procedure were explained verbally and written, and verbal informed consent was obtained for each child.
